# Confronting repressive ideologies with critical pedagogy in science classrooms

**DOI:** 10.1007/s11422-021-10047-7

**Published:** 2021-07-27

**Authors:** Wilton Lodge

**Affiliations:** UCL Institute of Education, 20 Bedford Way, Bloomsbury, London, WC1H0AL UK

**Keywords:** Critical consciousness, Dialog, Repressive ideologies, Equity, Critical pedagogy

## Abstract

The focus of this response to Arthur Galamba and Brian Matthews’s ‘Science education against the rise of fascist and authoritarian movements: towards the development of a Pedagogy for Democracy’ is to underpin a critical pedagogy that can be used as a counterbalancing force against repressive ideologies within science classrooms. Locating science education within the traditions of critical pedagogy allows us to interrogate some of the historical, theoretical, and practical contradictions that have challenged the field, and to consider science learning as part of a wider struggle for social justice in education. My analysis draws specifically on the intellectual ideas of Paulo Freire, whose work continues to influence issues of theoretical, political, and pedagogical importance. A leading social thinker in educational practice, Freire rejected the dominant hegemonic view that classroom discourse is a neutral and value-free process removed from the juncture of cultural, historical, social, and political contexts. Freire’s ideas offer several themes of relevance to this discussion, including his banking conception of education, dialog and conscientization, and teaching as a political activity. I attempt to show how these themes can be used to advance a more socially critical and democratic approach to science teaching.

The rise of ultranationalist far-right movements and support of fascist-related ideologies in the Western world have spurred a veritable discursive explosion around the role of the education system in fostering civic virtues of tolerance and respect for all sections of society (Crick, 2019). Contemporaneously, there has been ‘polyphony of voices’ (in Bakhtinean terms), such as Black Lives Matters (BLM), that has drawn attention to the systemic marginalization and institutional racial discrimination experienced by black, Asian, and minority ethnic (BAME) people within society and how these are reproduced and legitimized in the education system (Dabiri, 2020). Despite these interventions, evidence suggests that incidences of racism, Islamophobia, and xenophobia continue to proliferate within the school experience of BAME students and are deeply embedded in the education system. Recent statistics (Dabiri, 2020) and research (Runnymede, 2020) in the UK indicate that 95% of BAME students have witnessed or experienced racism at school and more than half of them think racism is the biggest obstacle to success. Moreover, researchers such as Louise Archer, Jennifer Dewitt, and Jonathan Osborne (2015) have also pointed to the near ubiquitous and deleterious practice of low expectations of black students by teachers. Such practices, they argue, may explain the underrepresentation and participation of BAME students in Science, Technology, Engineering, and Mathematics (STEM). Given these alarming data, scholars within the field of social justice (see, for example, Gillborn, 2014) have called for school curricula and discursive practices to reflect the diversity of contemporary society and mandate for engagement with anti-racist scholarship.

However, scholars such as Henry Giroux (2019) and Michael Apple (2005) have highlighted the inadequate commitment of policymakers in the USA in creating an inclusive and equitable curriculum which would challenge the institutionalization of fascist practices while promoting egalitarian principles and values. Such criticisms are not restricted to the USA, since in a recent survey, teachers in the UK lamented the lack of clarity about what a socially just curriculum should contain, and the lack of preparedness and training in tackling repressive ideologies in the classrooms (Parsons, 2019).

Similar concerns have also been raised in science education discourse (see, for example, Sheth, 2018). At the core of these debates is a recognition that science classrooms are repository sites of racialized injustices and structural oppression. Manali Sheth (2018) argues that while the science curriculum has been successful in promoting scientific literacy, it does not substantively engage with oppressive ideologies in science. Angela Barton (2001) echoes these views when she argues that because scientific literacy is seen as the major goal of science curriculums, issues such as the historical legacy of scientific racism are not confronted, and are seriously underemphasized.

These arguments of Sheth and Barton are especially important since it could be argued that the ideas invoked by ‘race science’ are still pervasive in contemporary public spheres, and invariably used to justify a race hierarchy. Shazia Absar (2020), for instance, observed that the pseudoscientific view that black people by virtue of having thicker skin than their white counterparts thus experience pain differently still persists in present-day health systems. Indeed, several recent studies have reported that black patients are less likely to be given pain medication than white patients (Wingfield 2020). In addition, Charles Murray and Richard Herrnstein’s (1994) specious arguments that black Americans were less intelligent than white Americans are simply extensions of the ideas of earlier prominent thinkers such as American and European scientists, including Thomas Jefferson, Louis Agazziz, Ernst Haeckel, and Samuel George Morton, to name a few. Indeed, Jefferson’s description of black people when he explained why emancipation was not possible without colonization is noteworthy:

The first difference which strike us is that of color … Whether it proceeds from the color of the blood, the color of the bile, or from that of some other secretion, the difference is fixed in nature …They seem to require less sleep … They are more ardent after their female: but love seems with them to be more an eager desire, than a tender delicate mixture of sentiment and sensation. Their griefs are transient. Those numberless afflictions, which render it doubtful whether heaven has given life to us in mercy or in wrath, are less felt, and sooner forgotten with them. In general, their existence appears to participate more of sensation than reflection ... Comparing them by their faculties of memory, reason, and imagination, it appears to me, that in memory they are equal to the whites; in reason much inferior … I advance it therefore as a suspicion only, that the blacks, whether originally a distinct race, or made distinct by time and circumstances, are inferior to the whites in the endowments both of body and mind. (cited from Hammond, Hardwick and Lubert, 2016, p. 24)

Undoubtedly, such examples of ‘race science’ had profound implications for black people around the world since race science was used not only as a justification for enslavement but to exclude them from self-governance. Sheth (2018) is firm in his belief that in order to disrupt these racial ideologies through science teaching, these ideas should be made explicit in the curriculum and students should be presented with data and works of scientists who have debunked the idea of race as a biological construct.

In his seminal work, *Science Education for Everyday Life*, Glen Aikenhead (2006) laments the failure of the school science curriculum to bring these issues to the core of its equity agenda. He blames this failure on the continued dominance of what he called the ‘pipeline ideology’ which emphasizes the paramountcy of scientific and technological knowledge for economic productivity and a reductionist science curriculum that continues to provide preparative education for the small percentage of young people who go on to pursue STEM-related careers. Aikenhead calls for a shift from this traditional, canonical science education to a more progressive humanistic approach where science is viewed as ‘culture’ and as a ‘social enterprise’. This perspective is akin to John Longbottom and Phillip Bulter’s (1999, p. 474) argument that “the principal justification for teaching science to all children is that it should make a significant contribution to the advancement of a more truly democratic society.” Thus, it is reasonable to argue that to achieve this goal, science educators must start from the assumption that grappling with repressive and anti-democratic ideologies is part of the business of science education.

Within this context, the argument advanced by Arthur Galamba and Brian Matthews in their paper ‘Science education against the rise of fascist and authoritarian movements: towards the development of a Pedagogy for Democracy’ (2021) is powerful, and raises important issues related to social justice and structural inequalities within science education. The paper builds on the work of scholars such as John Lawrence Bencze and Steve Alsop (2012), John Quicke (2001) and Alberto Rodriguez (2008), all of whom have contributed to the discourse about social justice in science education. The key point, around which their whole argument turns, is that science education has a social responsibility to empower students with agency and the critical tools to deconstruct and challenge fascist-related ideologies in order to make the world less intolerant, more egalitarian, less degrading and more humane. They call for the creation of an alternative science education curriculum that will allow scientifically literate people to think more ‘critically’, and propose a model for a ‘pedagogy for democracy’ that they believe will not only provide young people with logical science but also promote tolerance and inclusion. The authors examine, but only slightly, some of the arguments surrounding critical pedagogy and how this can be used as a counter-hegemony to neo-liberal, neo-conservative, and populist ideologies.

In what follows, I further explore how critical pedagogy can be used as a counterbalancing force against forms of discrimination within science classrooms. Locating science education within the traditions of critical pedagogy allows us to interrogate some of historical, theoretical, and practical contradictions that have challenged the field and to consider science learning as part of a broader struggle for justice and democracy (Giroux, 2010). Moreover, as noted by Henry Giroux (2010, p. 336), critical pedagogy allows students to “develop a consciousness of freedom, recognize authoritarian tendencies, connect knowledge to power and agency, and to challenge ideological and material forces that shape their consciousness.” In this pedagogical context, science learning is seen as a major vector in the process of social transformation.

My analysis draws specifically on the intellectual ideas of Paulo Freire, whose work continues to influence issues of theoretical, political, and pedagogical importance. A leading social thinker in educational practice, Freire rejected the dominant hegemonic view that classroom discourse is a neutral and value-free process removed from the juncture of cultural, historical, social, and political contexts. Freire’s ideas offer several themes of relevance to this discussion, including his banking conception of education, dialog and conscientization, and teaching as a political activity. I attempt to show how these themes can be used to advance a more socially critical and democratic approach to science teaching.

## Critical pedagogy and science education

Critical pedagogy, first articulated by Freire, is an interdisciplinary orientation inspired by ideologies that associate social theories to narratives of humanization. Its basic principles represent a range of radical ‘emancipatory’ educational ideas in which schools are seen as democratic spaces, producing learners who have developed critical agency and citizenry (Shor, 2019). Practitioners working in this tradition are committed to counter-hegemonic practices that challenge authoritarian ideologies while at the same time promoting equity and social justice in the classroom (Aronowitz, 2004). In this context, Freirean critical classrooms are not only a rejection of traditional ways of teaching but also an overt alternation of power relations and hegemonic structures, not only with teacher-student interactions but in the wider social context (Bizzell, 1991). Moreover, they present a repudiation of the anthropological notion of culture and the social reproduction of knowledge present in school curricula that support exploitation, oppression, and inequalities. Critical pedagogy, therefore, seek answers to the often-ignored questions: What knowledge should be selected for curricula? Whose knowledge matters? And whose knowledge is marginalized or excluded? What purpose does this knowledge serve? Such questions, though of importance to educational policy and practice, may not readily arise into the awareness of classroom practitioners and perhaps, as Freire (1990) points out, inadvertently contribute to the domestication of students.

Although criticized by some scholars for being too ‘abstract’, ‘utopian’, and based on rationalist assumptions that give rise to repressive myths (see, for example, bell hooks, 1994; Elizabeth Ellsworth, 1989), critical pedagogy seems well positioned to grapple with issues related to repressive ideologies in science classrooms. At the very least, if one accepts the notion of science as a social enterprise (as argued by Karl Popper, 2003 and Galamba and Matthews in their paper), there must be a recognition that scientific knowledge is formulated in processes of social contexts. In such contexts, as Popper (2003) maintains, scientific empirical data can be compromised by irrationality and biases of the scientist. There may also be a timorousness to accept ideas that contradicts their worldview (Kuhn, 1970). This view is authenticated by Popper (2003, p. 240) when he writes:

Everyone who has an inkling of the history of the natural sciences is aware of the passionate tenacity which characterizes many of its quarrels. No amount of political partiality can influence political theories more strongly than the partiality shown by some natural scientists in favour of their intellectual offspring.

Popper’s argument may well be the reason why scientists such as Richard Lyn, a self-proclaimed ‘scientific racist’, maintains, in spite of overwhelming evidence to the contrary, that Europeans have a higher average IQ than sub-Saharan Africans (Absar, 2020). Or why Nicholas Wade (2014) continues to claim that that race is an authentic scientific concept–that Darwin’s evolutionary theory has highlighted biologically distinct groups with genetically linked social attributes. Wade argues that the widespread incredulity at the concept of race as a biological reality is based on biased science, motivated by politically correct agendas. Tellingly, this argument has been repeated and perhaps authenticated by the Nobel Prize winner and geneticist, James Watson. The condemnation of Wade’s thesis was palpable and universal, with leading scientists whose work was cited in the book accusing him of hijacking and mispresenting their research to promote his racist agenda (Balter, 2014). David Dobbs (2014) in *The New York Times* suggested that not only are Wade’s views devoid of scientific evidence, but there is a suspicion that his motives are driven by prejudice against people of other races and a strong need to believe in their genetic inferiority.

The point that can be made here is that scientific knowledge developed in social contexts should foster healthy skepticism and a critical attitude towards claims of impartiality or definitive truth. However, this is not always the case as Popper (2003, p. 240; emphasis in original) acknowledges “… [there] is an astounding failure to understand precisely … the *social aspects of* [scientific] *knowledge*.” Galamba and Matthews in their paper call for the curriculum and science discourse to make explicit the social context in which science operates and further warn that if this is not done, their remains a danger that students will have an idealized view of science–that science is rational and value-free. Instead, students need to understand that science is fluid, shifting, tentative and debated.

A Freirean classroom, as Giroux (2009) relates and Rodriquez (2008) elaborates, seeks to fill this dearth by providing an intellectual space in which students are given freedom to examine the complex interplay of the social, cultural, emotional, and intellectual dimensions underpinning scientific discursive practices. In such a liberating classroom, students are engaged in an open narrative to interrogate the nature of science and, indeed, to question what counts as ‘normal science’, and the diverse and complex value systems that scientists bring to their work. The ultimate goal of this approach aligns with the boarder struggles for justice and democracy as articulated by Freire (1990). What is more, students develop critical science agency which helps them to “develop consciousness of freedom, recognize authoritarian tendencies, and connect knowledge to power and the ability to take constructive action” (Giroux, 2010, p. 336).

## Banking conception of education

Arguably, one of the most generative themes in Freirean pedagogy is the banking concept of education. In this formulation, learners are perceived as passive and anti-intellectual objects to be filled with knowledge and are lectured into ‘sleepy silences.’ Freire (1990, pp. 72–73) writes:

Education thus becomes an act of depositing, in which the students are the depositories, and the teacher is the depositor. Instead of communicating, the teacher issues communiques and makes deposits which the students patiently receive, memorize, and repeat ... [Thus] knowledge is a gift bestowed by those who consider themselves knowledgeable upon those whom they consider to know nothing ... The more students work at storing the deposits entrusted to them, the less they develop the critical consciousness which would result from their intervention in the world as transformers of that world.

Implicit in this quotation from Freire is the view that locates the banking concept of education model within the broader debate of power and domination in the education system. My conceptualization of power here is consistent with Lukes’ (2005) articulation in which people’s interests are contorted through ideological hegemony. This position is captured by Freire (1990, p. 73) himself in his meticulous catalogue of the tenets of the banking concept model. Alongside the usual characteristics of a traditional classroom he compares the teacher’s domination of the learning process to a subject-object relationship in which opportunities for any forms of inquiry-based learning are limited. This, he argues, affirms “an absolute ignorance onto others, a characteristic of the ideology of oppression, negates education and knowledge as processes of inquiry” (Freire, 1990, p. 72). Moreover, the learner’s ability to engage in critical thinking is reduced and they simply are expected to repeat verbatim the teacher’s perception of what constitutes true knowledge of the social world (Rodriquez, 2008). As a consequence, learners internalize values and habits and “learning becomes the ingestion, or appropriation, of specified gobbets of intellectual capital; by some process of alchemy, knowledge simultaneously belong to all” (Yandell, 2017, p. 249).

Freire was particularly critical of the teacher’s role in banking education. Essentially, he saw their role as functionaries of the hegemon (in Gramscian terms), exercising ultimate power over their students as they formulate and disseminate organic ideologies. Giroux (1988), through his reconceptualization of Freire’s work, suggests that in order to retain control, teachers need to sustain a monologic discourse that negates any form of free inquiry in the classroom. In such classroom climates, students internalize these repressive values which incapacitate their critical thoughts.

Freire counterposes the banking conception with the ‘problem-posing’ approach, in which teachers and students raise questions about the social production of knowledge. Moreover, as observed by Ira Shor (2019), in such Freirean classrooms, the relationship is reciprocal, insofar as both teachers and students are willing to search and construct knowledge while developing egalitarian and democratic values in the process. The problem-posing approach demands that teachers provide a platform for students to develop an authentic voice in challenging neo-liberal ideas, while promoting social actions.

The notion of the banking model has opened up an intellectual space for researchers to examine pedagogies in science classrooms, and the extent to which they are able to address the social realties in which children live (Rodriguez, 2008). In banking approach classrooms, as stated above, students are seen as passive entities and the experiences that they bring to the learning process are ignored, including those linked to their class, gender, and racialized positions. The teacher is free to promote a monocultural of understanding of science that is often closely associated with positivist views of science (Elshof, 2014). Consequently, students acquire a naïve view of the nature of science and are left struggling to understand how science learning connects to their lived experiences. This may, in part, explain why so many BAME students are of the opinion that science is not for them (Archer, Dewitt and Osborne 2015).

Further questions have been raised by scholars such as Jonathon Osborne and Sara Hennessy (2007) about the efficacy of the banking model paradigm in science classroom in so far as it is able to develop critical thinking. Critical thinking is seen not just as an essential aim of science education but an important skill for democratic citizenship, since it is related to the values of rationality and reason. As Longbottom and Butler (1999, p. 489) acknowledge:

Citizens who are critically minded, and who can analyze and challenge social structures, will be able to implement democratic ideals. In this way, science education, in combination with a general education that teaches democratic ideals, can play a valuable part in equipping citizens with knowledge for action.

The crucial point that Osborne and Hennessy (2007) raise is that if students are coerced into acquiescing with the prevailing view of the purpose, nature, and role of science, there will be limited opportunities to develop their critical reasoning skills. Students may therefore lack the ability to engage in a reasoned argument about scientific issues that contribute to an unjust social order.

## Science teaching as a political activity

Many of the central concepts of critical pedagogy are crystallized in Freire’s (1990) seminal work *Pedagogy of the Oppressed*, first published in Portuguese in 1968 and described by Giroux (2010) as one of the most influential books of the twentieth century, in which Freire offers a critique of the oppressive elements of the education system. In this assessment he questioned the notion of neutrality in the education system, writing:

There is no such thing as a neutral education process. Education either functions as an instrument that is used to facilitate the integration of the younger generation into the logic of the present system and bring about conformity to it, or it becomes ‘the practice of freedom’, the means by which men and women deal critically and creatively with reality and discover how to participate in the transformation of their world. (Freire 1990, p. 34)

Freire’s argument here is persuasive and has particular relevance for the teaching of school science, since the extent to which scientific knowledge and science education are value-neutral domains is still fiercely debated (Donnelly, 2002). An example of this ‘uneasy relationship’ can been seen in the central role that governments and industries continue to play in certain fields of scientific research, such as climate science, weapons research, embryonic stem cell research and, more recently, in the development and deployment of COVID-19 vaccines. Even if one accepts that such support for these scientific endeavors comes from a belief that a positive outcome will improve the economic and social wellbeing of the wider society, there lies a danger that science itself can be tainted by ideology. Take, for instance, the US government role in the Tuskegee syphilis experiment which denied hundreds of black men treatment for the disease in order to study its progression, despite the availability of such treatment. Or President George Bush’s decision to terminate federal government funding for embryonic stem cell research in the United States, choosing instead to support research on umbilical cord placenta and animal stem cells. In justifying this decision, Bush declared that “my position on these issues is shaped by deeply held beliefs” (Bush 2001, para 21). Likewise, the Trump administration’s decision to roll back climate-related funding by questioning the science surrounding climate change and dismissing it as a hoax created by China (Kormann, 2016). These examples support Michael Reiss’s (2003, p. 155) contention that “values are inevitably and inexorably conflated with science.”

More recently, the entanglement of politics and science played out spectacularly in the public sphere, when Boris Johnson, the UK Prime Minister, intervened to prevent government scientific advisors from responding to questions concerning the public health ramification of breaking lockdown rules by the then chief political advisor, Dominic Cummings. Critics questioned the politicized role of the government scientific advisory board, accusing them of being complicit and dangerously collusive in their blind support of government policy. Indeed, such was the fallout that headlines such as ‘The silence of chief scientists is worrying and deeply political’ (*New Statesman*, 2020) and ‘Public trust in science at risk’ (*The Guardian*, 2020) have become common in the UK media. At the very least, such criticisms may suggest that science can no longer viewed “as an autonomous realm of rationality, of unbiased search for truth and knowledge conducted by disinterested people of the highest ethical standards” (Brown and Malone, 2004, p. 116).

Yet, despite the above observations, as Parsons (2019) argues, school science is still normatively presented to students without context, appearing as rational, unbiased, and neutral, divorced from human inconstancy and ideology. However, such a mythopoeic representation obscures the political and social context from which scientific knowledge is formulated. The implication here is that students are offered a naïve view of science.

## Dialog and conscientization

Another key contribution of Freirean pedagogy is the notion of conscientization. In his (1973) *Education for Critical Consciousness*, he defines conscientization “as the learning to perceive social, political and economic contradictions, and to take action against the oppressive elements of reality” (p. 35). To put it more succinctly, conscientization is the process of acquiring critical consciousness that has the power to transform one’s social reality. Freire (1990) argues that it is only by developing students’ critical consciousness will they feel empowered to challenge existing undemocratic ideologies, to reflect upon their own biases and assumptions, and to act to change the situation around them. For Freire, developing critical consciousness is crucial to producing transformative potential and helping individuals reclaim their humanity from the shackles of unjust systems. In support of Freire, Giroux (2009) argues that if people’s critical consciousness is awakened, then they will be able to effect change in their communities by analyzing and challenging the social structure. A similar perspective is also suggested by Shirley Steinberg and Joe Kincheloe (2001) when they contend that critical consciousness raising is not just a means of identifying prevailing hegemony and the root causes of oppression but should also empower people to participate in socio-political actions and other organizational engagements. This argument is very similar to the one made by Galamba and Matthews about the need for science education to engage young people in social activism.

Ira Shor (1996) emphasized three core constructs which are significant for developing critical consciousness: critical reflection, political efficacy, and critical action. Critical reflection, he argued, is central to the relationship between theory and practice, and requires the individual to recognize and interrogate deeply held assumptions concerning issues related to inequalities. Political efficacy refers to the belief that individuals can play an active role in producing political and social reforms and is seen as essential for active citizenry in a stable and modern democracy (Schulz 2005). Critical action results when an individual, having become aware of his or her agency, begins to change their unjust conditions through socio-political actions. These constituents are thought to be established in phases of transience thoughts – from intransitive (people feeling a sense of hopelessness and being resigned to their fate) to semi-transitive (people develop some awareness of their situation and want social change) to critical transitive thoughts (people think thoroughly and see themselves as agents of change) (Shor 1996).

Equally important in relation to Freire’s conceptualization of critical pedagogy is the notion of dialogical practice which Freire argued was an existential necessity for recovering the voice of the oppressed (Freire 1997). According to Shor and Freire (1987), dialog is not only a tool for promoting cognitive learning or to develop self-esteem, but it is a means to empower young people to confront social injustices as they work towards emancipation and social transformation in the classroom. In so doing, relational opportunities are established as ‘dialoguers’ search together for truth while at the same time developing consciousness-raising and progressive values. This process is positioned in the speech, language, and social realities of the learners and comprises a compelling illuminating moment in which hegemonic structures are brought into focus and political engagement is imperative.

Freire (1990, p. 92), however, emphasizes that “true dialogue cannot exist unless the dialoguers engage in critical thinking … thinking which perceives reality as process, as transformation, rather than as a static activity.” What is more, as Freire (1990) goes on to argue, genuine dialog cannot be imposed or deposited on individuals. It is, by its very nature, a cooperative activity characterized by respect and tolerance.

If one accepts Longbottom and Bulter’s (1999) and Quicke’s (2001) arguments that a major goal of science education is, or should be, the advancement of equality and social justice to unlock the inherent humanity of its citizens, then the development of students’ critical consciousness becomes paramount. Pedagogically, this means a radical shift from a curriculum heavily weighted towards ‘the pipeline ideology’ of traditional science teaching to a more progressive one that empowers students to challenge the existing social structure, to reflect upon their own biases and assumptions, and to act change the situation around them. This is somewhat akin to what Longbottom and Butler (1999) describe as ‘a knowledge for action education’.

Establishing democratic dialog is essential to the development of critical consciousness. Since the ultimate goal is to achieve equality and democracy, teachers should not detach themselves from the discourse. According to Freire (1990), they have the right to present their own personal views of the subject under inquiry. Thus, if, for example, a science teacher holds racist, sexist, fascist, or homophobic views, they should be allowed to present these ideas (subject to legislative requirements and school regulations), but should not impose them on the learners. Instead, they should present them inside a thematic narrativization, and ensure that students are given opportunities to interrogate and reject the teacher’s perspective. This intricate balance between the learners and teachers is central to instilling democratic practices as well as critical ideas.

## Concluding remarks

The main goal of this paper is to make the argument for and explore, as part of the wider debate on social justice in science education, how critical pedagogy can be used as a counterpoising force against forms of discrimination within science classrooms. Although Freirean ideas have been debated in science education scholarship for many decades, they have yet to be given a prominent place in science curricula and classrooms. Scholars such as Arturo Rodriquez (2008) have blamed this on the continued influence of neoliberalist ideologies within school science, which emphasize the production of future scientists for the corporatocracy. However, as I have argued, a science education that is fit for purpose in the twenty-first century must take seriously its role in tackling racialized injustices and structural oppression while promoting democratic ideals within the classroom. It is here that Freire’s educational ideas provide fruitful grounds for science education. For it is only with the establishment of a dialectic process that is committed to the development of critical consciousness that students will have an authentic understanding of how science can be used to advance repressive ideological agendas.

Adopting a Freirean approach in science classrooms will undoubtedly pose distinctive challenges for science teachers. Indeed, many science teachers might subscribe to Maxine Hairston’s (1992) assertion that critical pedagogy is ‘naïve’ and ‘self-servicing’, an approach which provides a platform to indulge one’s political bias without consequences. Others may hold the view that a science education that puts social and political ideologies at the centre of science teaching will limit students’ understanding of key scientific concepts. While these are fair points to consider, what is clear is that if school science is to make a meaningful contribution to democratic fairness and social change, then science educators will need to draw from a number of critical perspectives, including the intellectual ideas of Freire.
